# 40 Arthralgia and celiac disease

**DOI:** 10.1093/rheumatology/keac496.036

**Published:** 2022-10-07

**Authors:** N Boutrid, H Rahmoune, B Bioud, M Amrane

**Affiliations:** 1 LMCVGN Laboratory, Faculty of Medicine, Sétif-1 University; 2 Pediatrics, Setif University Hospital

## Abstract

**Background:**

In a large cohort of 2125 children followed in rheumatology, 2% had celiac disease (CD) (about 3 times the rate in the general population). Inversely, CD was reported in up to 2–3% of children with juvenile idiopathic arthritis

**Objectives:**

Reporting joint damage such as arthrlagia in a celiac pediatric cohort

**Material and method:**

We collected 58 celiac children and reported those with arthralgia and studied their clinical aspects

**Results:**

Arthralgia was reported in 10/58 children (i.e. 17.24%) of the entire cohort studied (*n* = 58), including 09 children with positive HLA DQ2/DQ8 typing, potentially with an autoimmune background. The arthralgia was not accompanied by arthritis, and had an apyretic, labile and transient character. Only one case of episodic spinal pain was reported in the history

**Conclusion:**

More than a sixth of the pediatric celiac cohort presents with arthralgia. These joint pains can be caused by vitamin D deficiency, osteoporosis, associated juvenile idiopathic arthritis (JIA), in particular HIV-positive. They can also be isolated and respond to the gluten-free diet.

